# Insights into Diversity and Imputed Metabolic Potential of Bacterial Communities in the Continental Shelf of Agatti Island

**DOI:** 10.1371/journal.pone.0129864

**Published:** 2015-06-11

**Authors:** Shreyas V. Kumbhare, Dhiraj P. Dhotre, Sunil Kumar Dhar, Kunal Jani, Deepak A. Apte, Yogesh S. Shouche, Avinash Sharma

**Affiliations:** 1 Microbial Culture Collection, National Centre for Cell Science, Pune, 411007, Maharashtra, India; 2 Conservation Department, Bombay Natural History Society, Hornbill House, Mumbai, 400 001, India; CAS, CHINA

## Abstract

Marine microbes play a key role and contribute largely to the global biogeochemical cycles. This study aims to explore microbial diversity from one such ecological hotspot, the continental shelf of Agatti Island. Sediment samples from various depths of the continental shelf were analyzed for bacterial diversity using deep sequencing technology along with the culturable approach. Additionally, imputed metagenomic approach was carried out to understand the functional aspects of microbial community especially for microbial genes important in nutrient uptake, survival and biogeochemical cycling in the marine environment. Using culturable approach, 28 bacterial strains representing 9 genera were isolated from various depths of continental shelf. The microbial community structure throughout the samples was dominated by phylum *Proteobacteria* and harbored various bacterioplanktons as well. Significant differences were observed in bacterial diversity within a short region of the continental shelf (1–40 meters) i.e. between upper continental shelf samples (UCS) with lesser depths (i.e. 1–20 meters) and lower continental shelf samples (LCS) with greater depths (i.e. 25–40 meters). By using imputed metagenomic approach, this study also discusses several adaptive mechanisms which enable microbes to survive in nutritionally deprived conditions, and also help to understand the influence of nutrition availability on bacterial diversity.

## Introduction

Marine environment is a diverse habitat harboring wide range of organisms with abundant metabolic potentials. Continental shelves are known to contribute largely to the total carbon and nitrogen cycling in the marine ecosystem [1,2]. Previous oceanographic studies focusing on carbon productivity, suggest that there is a high rate of carbon transfer from the shelf to the deep ocean, either through physical or biological processes [2]. Almost all the metabolic activities carried out in the ocean have been observed to be mediated by microorganisms [1,3]. Most of this microbe driven ocean biochemistry is a part of their own metabolism specialized for substrate utilization and survival in the marine environment, which eventually is a part of a larger pathway of global biogeochemical cycle [1–3]. This includes various substrate sequestering and homeostasis mechanisms in nutrient depleted and other extreme zones present in the marine environment [3,4]. Many reports suggest that, marine sediments are richer in microbial diversity than the upper water column [5–7]. Availability of high amount of biomass in upper water column is considered as a probable reason for high microbial diversity in the sediments; which is due to degradation of dead organisms settling down and providing ample nutrients to the microbial community inhabiting in sediments [7]. Considering wide potential of microbes dwelling in the marine environment, it is indeed significant to determine the microbial community structure and their metabolic potential. In this study, we examined bacterial diversity of sediments from the continental shelf of the Agatti Island, Lakshadweep Archipelago.

Lakshadweep islands are located in the Arabian Sea (Indian ocean) between latitude 8° and 12° 30’ N and longitude 71° and 74’ E at a distance ranging from 200–400 km from the mainland (coastal region of Kerala). There are in all 36 islands, which are invariably surrounded by huge and shallow lagoons at the western side [8,9]. The morphologic location of the Indian ocean is unique from other oceans, as it is surrounded completely by land in the northern side and it doesn’t extend in low temperature zones of the northern hemisphere [10]. Thus, a lot of dynamic atmospheric and oceanic circulations are seen in this region, as a result of contrasting north eastern (NE) and south western (SW) monsoon periods [10,11]. Observations suggest that along with primary production in the ocean, concentration of nutrients and oxygen, are affected by these seasonal variations [10,11].

Present study attempts to explore microbial diversity of the continental shelf from Agatti Island, along with *insilico* prediction of their functional capabilities. The sediment samples were collected from various depths of the continental shelf. A combined approach of deep sequencing and culturable technique was employed to study this imperative biogeochemical hotspot.

## Methods

### Sample collection and community DNA extraction

The sediment samples were collected at various depths from the continental shelf region of the Agatti island, (10° 52' 47.32"N, 72° 10'11.86"E), ranging from 1 meter to 40 meter. In total, 10 samples were collected (Table 1), stored aseptically in sterile (gamma-irradiated) containers and were transported to the laboratory at 4°C. The aliquots of samples were then subjected to DNA extraction using MoBio power soil kit (MoBio, USA) as per the manufacturer’s instructions. The quality of DNA was checked on 0.8% agarose gel and concentration was measured using Nano Drop ND-1000 spectrophotometer (Nano Drop technologies, Willingminton, USA). The DNA was stored at -20°C until further processing.

**Table 1:** Sequence summary and alpha diversity indices. The table illustrates the sequencing depth along with the diversity indices calculated for each sample.

sample	Raw reads	Quality reads	Assigned reads	Observed Species	Chao1	Shannon	Simpson	Simpson reciprocal
**SG01**	406962	405478	337915	247	485.6363636	0.309425842	0.050559601	1.053252001
**SG02**	494301	492261	393878	428	706.7222222	2.789304729	0.637811543	2.760993566
**SG05**	445440	443609	344017	758	1159.072917	4.641690631	0.917812088	12.16723937
**SG10**	560284	557402	276379	1151	1820.754717	4.328062536	0.768416938	4.318105094
**SG15**	574266	569861	236313	1639	2577.044693	5.737905279	0.933913217	15.13161864
**SG20**	400659	398889	323046	742	1178.8	4.407034847	0.895815914	9.598394946
**SG25**	570756	567372	320983	1084	2003.820896	3.396951651	0.729327616	3.694503247
**SG30**	348251	346325	117888	1978	3419.33195	6.632792849	0.970732214	34.16725759
**SG35**	471048	469058	251788	1532	2508.96875	5.894616632	0.955063637	22.25369241
**SG40**	380889	379080	293268	508	1107.842105	3.501956883	0.849711064	6.653849777
**Total**	**4652856**	**4629335**	**2895475**	-	-	-	**-**	**-**

### Isolation and identification of bacteria

The aliquots of sediment samples were serially diluted (10^−1^ to 10^−6^) and suspension was spread on different media plates as per the standard spread plate technique, used for isolation of bacteria. Various growth media like Sea Water Agar, R2A, Zobell Marine Agar, Nutrient Agar, Luria Agar, Tryptone Yeast extract Agar (pH = 7.2 ± 0.2) were used and plates were incubated at 30°C for 48 hours. The isolates were purified by re-streaking of isolated single colony and were preserved in glycerol stocks in deep freezers (-80°C). Simultaneously, bacterial isolates were identified using 16S rRNA gene sequencing using universal bacteria specific primers (27F: 5’GAGTTTGATCMTGGCTCAG-3’ and 1492R: 5’-TACGGYTACCTTGTTACGA-3’) as described in previous reports [12]. Sequencing was carried out using 96 capillary DNA Analyzer 3730XL (Applied BioSystems, USA) as described in previous reports. [13]. The sequences obtained were quality checked, trimmed and assembled with DNASTAR SeqMan Pro v10 and later identified using EzTaxon database [14]. Sequence similarity for each sequence was obtained and is shown in S1 Table. Additionally, phylogenetic analysis of these sequences was carried out as described by Prakash *et al*. (2014).

### Amplicon sequencing of bacterial 16S rRNA genes

The total DNA extracted from each sample was checked for its quality and concentration. DNA templates (concentration >50 ng/μl) were used to amplify the V3 region of the 16S rRNA gene [15]. Template and library preparation was carried out according to the manufacturer’s protocol (Illumina, USA). The sequencing of multiplexed 16S rDNA amplicon libraries was performed on Illumina Miseq platform using paired end 2 x 150 bp chemistry.

### Pre-processing and sequence data analysis

The raw paired end reads obtained were assembled using PANDAseq (Paired end assembler for DNA sequences) tool [16]. Default options available in the tool were used to obtain assembled sequences. If a mismatch or ambiguous base calls were observed, the paired-end sequences involved in the assembly were discarded. Sequence reads were assigned to operational taxonomic units (OTUs) in QIIME v1.7 [17] by using a closed reference-based OTU picking approach with Greengenes database (release May 2013) [18]. The OTU picking was carried out using UCLUST method with similarity threshold of 97%. [19]. Taxonomic assignments were performed using RDP naïve bayesian classifier [20] in MOTHUR v1.25 [21]. Data was normalized according to the least number of reads per sample (i.e. 1,17,888 reads in sample SG30) before performing further analysis. Alpha diversity indices and beta diversity plots were also obtained using QIIME v1.7. For beta diversity analysis, Bray-Curtis distances were calculated from the table of OTU counts. Significant differences in the OTUs at different taxonomic level and different depth were evaluated using statistical tests like ANOVA (Tukey’s HSD) and Adonis test. Additionally, on the basis of classified sequences, we determined imputed metagenomic functions like metabolic capabilities of the bacteria at various depths using PICRUSt: phylogenetic investigation of communities by reconstruction of unobserved states [22] and differences were evaluated using t-test.

### Nucleotide sequence submission

The sequences obtained from high throughput sequencing effort, were submitted to NIH database using the sequence read archive (SRA) submission with the accession number SRP043450. 16S rRNA gene sequences of the isolates were submitted to NCBI GenBank database under the accession numbers: KM037727- KM037754 and KF732820.

### Ethics statement

No specific permissions were required for this study, as no human subjects were involved and also did not include any endangered or protected species.

## Results and Discussion

### Culturable bacterial diversity

**Fig 1:**
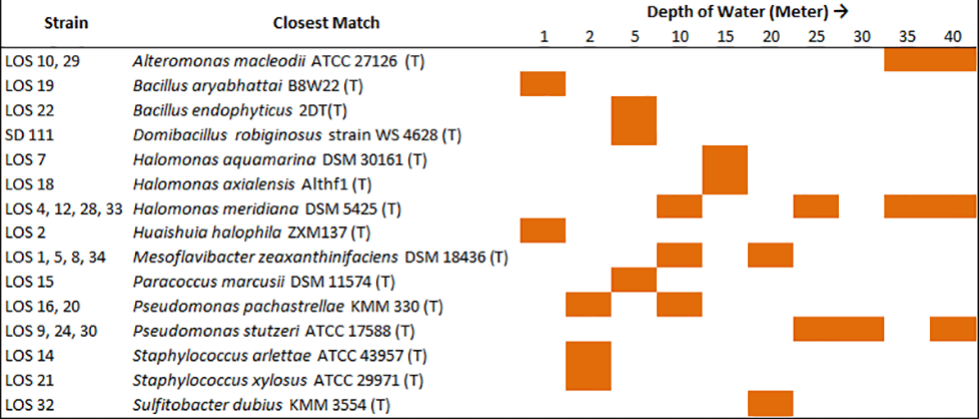
Spatial distribution of bacterial isolates. The figure depicts the spatial distribution of the representative bacterial isolates across the sampling depths.

Spread plate technique used for isolation of bacterial strains from sediment samples resulted in isolation of 28 different bacterial strains from various depths as described in S1 Table. The taxonomic identification was done by 16S rRNA gene sequencing, which demonstrated the presence of 9 different genera (S1 Table). The phylogenetic analysis was carried out using sequences of closely related bacterial type strains from EzTaxon database, which also substantiated the taxonomic identification (See S1 Fig). The Phylogenetic tree analysis showed that most of the isolates formed two major clades of *Gamma* and *Alpha-Proteobacteria*, while some of the other bacterial isolates belonging to *Firmicutes* and *Cytophaga-Flavobacterium-Bacteroides* (CFB) group of phyla formed distinct clades within the tree, thus indicating the presence of diverse bacterial group in the habitat. Fig 1 depicts the spatial distribution of the bacteria isolated from various depths. Genera with diverse functional capabilities were observed at various depths. The genus *Halomonas* was observed to be widely distributed across the depth (10, 15, 25, 35 and 40 meters). These bacteria are known to be halophiles or are extremely halotolerant like *Halomonas meridiana* and thus could be a probable reason for their ubiquity in the marine environment [23]. The xylanase producing bacterium *Mesoflavibacter zeaxanthinifaciens* was also found ubiquitously in sediment samples (see Fig 1). This bacterium utilizes xylan, which is one of the major polysaccharides in plant cell wall [24,25]. The continental shelves harbor diverse population of marine plants, which may serve as a source of nutrition to such bacteria [26]. Other bacteria isolated like *Huaishuia halophila* were previously reported to be associated with algal blooms in the coastal regions [27]. *Huaishuia* produces *alginase*, which has a specific function of utilizing alginate, a polysaccharide found in the cell wall of the brown algae (*Phaeophyceae*) [27,28]. Additionally, the bacterium *Alteromonas macleodii* isolated from sediment samples is known to be widespread in the tropical environments as an opportunistic copiotroph harboring the Na^+^/H^+^ antiporters to survive in high salinity environments [29,30]. We also found a novel bacterium *Domibacillus indicus*, which was isolated from the depth of 5 meters [31]. This bacterium showed 97.6% 16S rRNA gene sequence similarity with the only other species reported, i.e. *Domibacillus robiginosus* strain WS 4628^T^, in genus *Domibacillus* [31]. We were also able to isolate the bacterium *Sulfitobacter dubius* at the depth of 20 meters. This bacterium is previously reported to be isolated from sea grass (*Zostera marina*) and most of the species belonging to genera *Sulfitobacter* are reported to be heterotrophic bacteria residing in hypersaline environments [32]. Furthermore, *Sulfitobacter* is one of the groups of bacteria which form the *Roseobacter* clade [33]. The *Roseobacter* lineage in the marine habitat is known to be conducive to the cycling of oceanic carbon by producing ABC (ATP-binding cassettes) importers and exporters [34]. Additionally, other genera which are ubiquitously found in various ecological niches were also found at various depths as listed in S1 Table.

### Diversity and species richness of uncultured bacterial communities

The high throughput sequencing effort yielded a total of ~4.65 million reads with an average of 4,65,285 reads per sample. After assembly and quality assessment of the paired end reads, a total of ~4.62 million high quality reads (4,62,933 average reads per sample) were obtained, which accounts up to ~99.4% good quality usable reads (Table 1). Taxonomic assignment with reference database could be done to ~2.89 million reads (2,89,547 average reads per sample) at similarity threshold of 97%, and were used for further analysis. After taxonomic assignment, out of 6,943 OTUs obtained for all samples, 5,491 rarified OTUs were used. The average Good’s coverage for all the samples was found to be 99.65% ± 0.001% (mean ± SD) indicating that majority of the bacterial diversity was captured. Additional alpha diversity estimators and observed species are shown in Table 1. We also calculated the richness using some Non-parametric estimators like Chao1, which was observed to be highest (3419.33) for the depth of 30 meters. Additionally, the Shannon index was also found to be highest (6.63) for the sample collected at the depth of 30 meters. The diversity indices calculated across the depths of continental shelf revealed that the overall bacterial diversity was less in the upper most regions i.e. 1–5 meters and more in the higher depths (Table 1).

**Fig 2:**
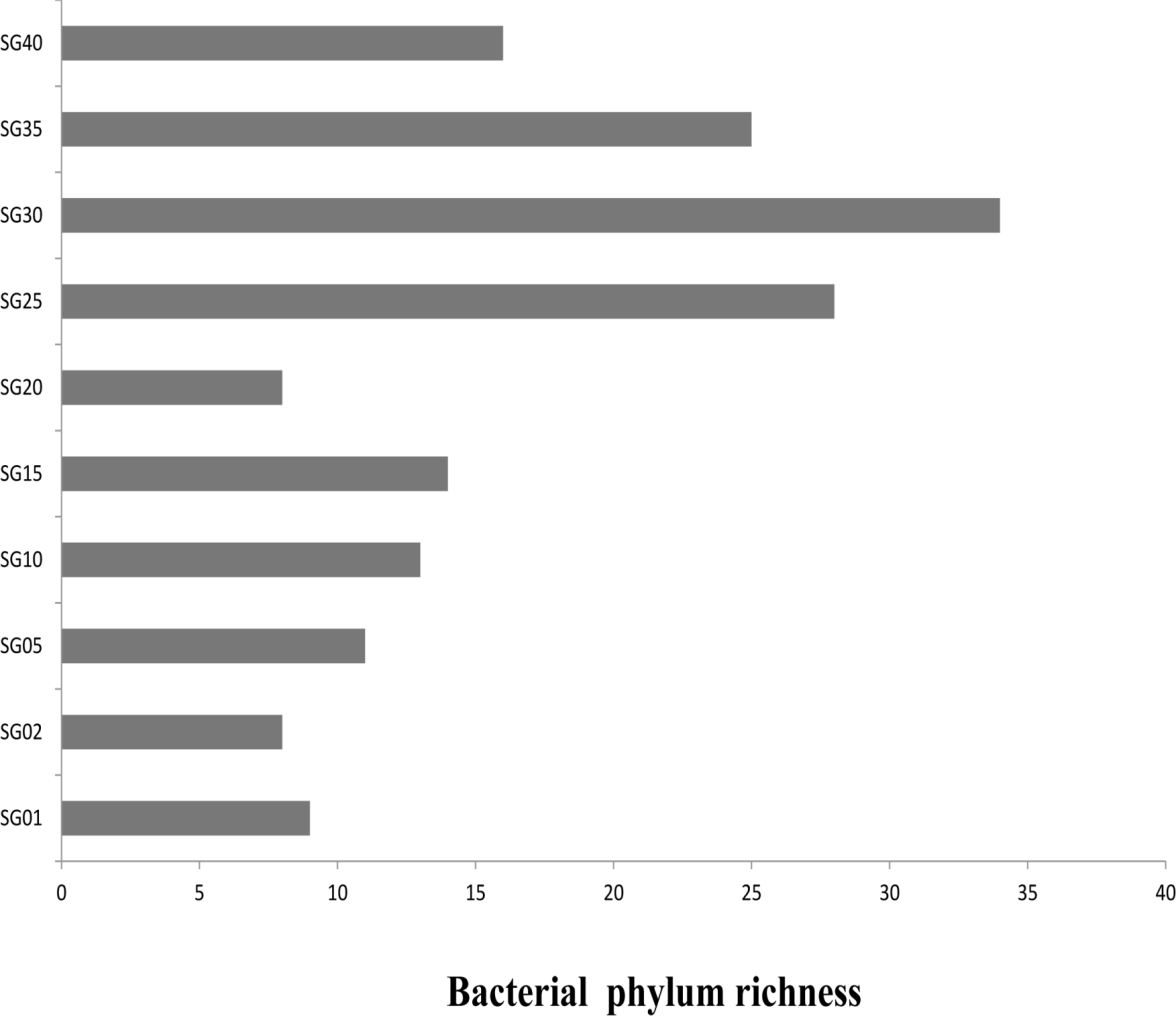
Phylum level bacterial richness at various depths. The X-axis represents the number of phyla (sum of the values given for presence/absence (binary values) of the particular phylum at respective depth), while Y axis represents the samples.

**Fig 3:**
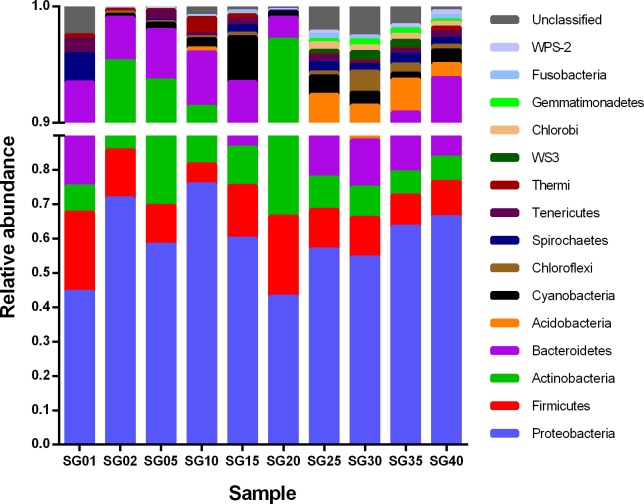
Phylum level bacterial diversity across various depths. The diagram depicts the distribution of the first 15 dominant phyla identified across the samples. The Y-axis represents the percent relative abundance of each phylum. A break is introduced on Y-axis at 0.89 to resolve the Y-axis within the range of 0.9–1.0.

Furthermore, bacterial richness was observed to be highest at the depth of 30 meters. Bacterial richness at phylum level was found to be more at higher depths (Fig 2.). A total of 15 phyla were observed in the samples collected from various depths. The first 5 abundant phyla contributed up to 95.29% of the total bacterial diversity (S2 Table). The total bacterial community analysis showed that the phylum *Proteobacteria* was most dominant, contributing up to 59.49% ± 10.56%, followed by *Firmicutes* (13.52% ± 5.71%) and *Actinobacteria* (12.51% ± 8.13%). Among *Proteobacteria*, *Gammaproteobacteria* and *Alphaproteobacteria* contributed the most, which have developed several mechanisms like nutrient transport systems (ABC: ATP-Binding Cassettes and TRAP: Tri-partite ATP-independent periplasmic transporters), oligotrophic and chemoautotrophic growth mode to survive in the marine environment and thus could be the probable reason for being one of the most dominant bacterial phyla in the marine habitat [3,5,35,36]. Other than *Proteobacteria*, phyla like *Actinobacteria*, *Firmicutes* and *Bacteriodetes* were also found in all depths (Fig 3). Occurrence of *Cyanobacteria* in sediment samples indicates presence of photosynthetic activity in the continental shelf along with other bacterioplanktons. Additionally, bacteria belonging to families like *Flavobacteriaceae* and *Rhodobacteriaceae* were observed in the sediment samples from higher depths (Table 2). These bacterial families were previously reported to be associated with algal blooms [37] and are known to utilize polysaccharides as substrates from algae [3,4,37]. Some reports suggest that family *Rhodobacteriaceae* consists of many phototrophs [38,39], which may also play a key role in total photosynthetic activity in the continental shelf.

**Table 2:** Statistical test (ANOVA tukey) results for the phylum and family level diversity between UCS and LCS.

**Phylum**	**Tukey's HSD test (p>0.05)**
*Thermi*	0.352178565
*Acidobacteria*	0.000100923*
*Actinobacteria*	0.163781512
*Bacteroidetes*	0.124942764
*Chloroflexi*	0.034693327*
*Cyanobacteria*	0.866290178
*Firmicutes*	0.204432483
*Proteobacteria*	0.851119077
*Spirochaetes*	0.773538214
*Tenericutes*	0.894531755
**Family**	**Tukey's HSD test (p>0.05)**
*Flavobacteriaceae*	1.716E-06*
*Rhodobacteraceae*	4.92E-06*
*Sphingomonadaceae*	0.024973404*
*Alcaligenaceae*	0.020638087*
*Pseudomonadaceae*	0.058554691
*Vibrionaceae*	0.00510619*

* indicates significant values

### Comparison of bacterial communities at various depths

**Fig 4:**
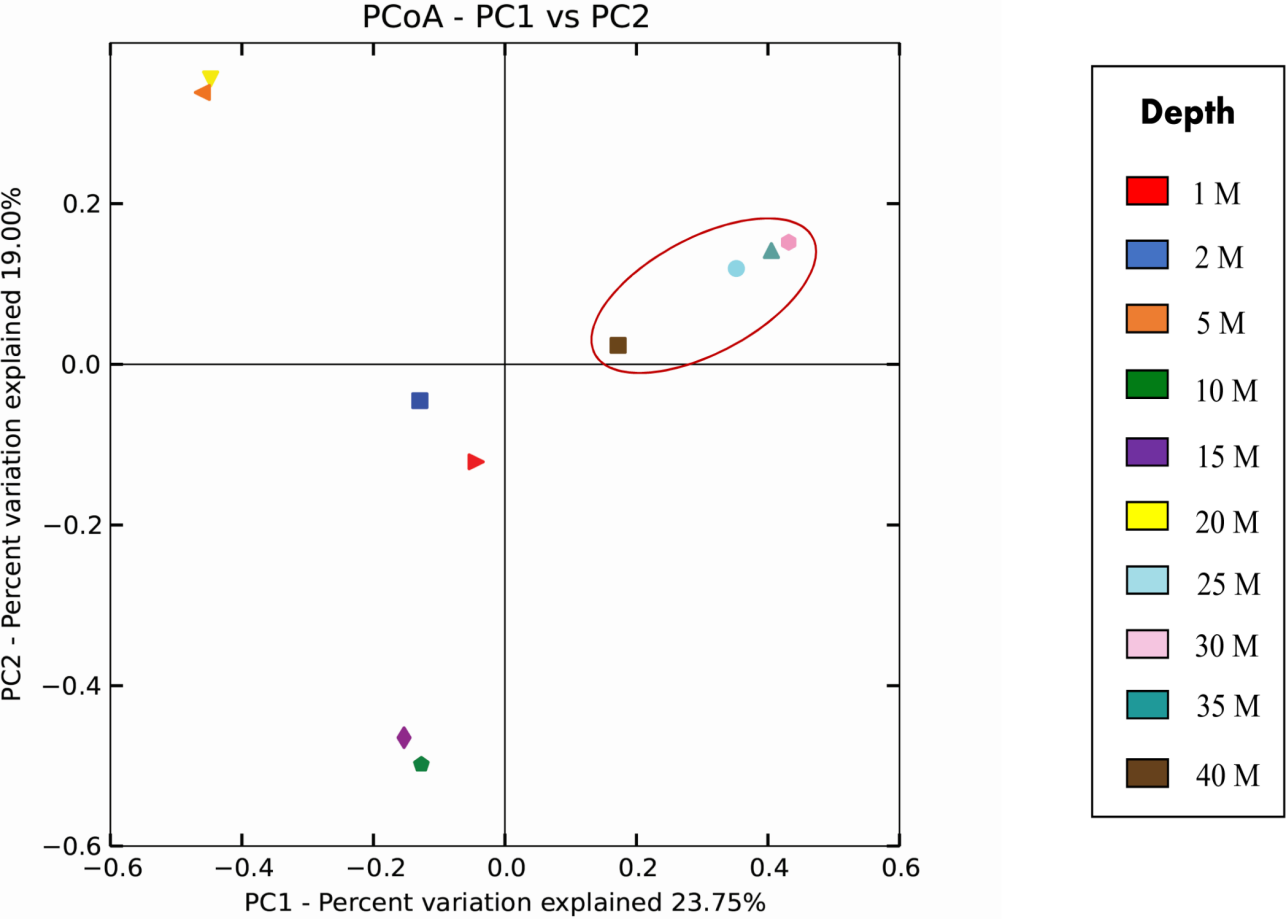
PCoA analysis. The plot was constructed based on the Bray-Curtis distance between the samples obtained using weighted unifrac analysis. The analysis elucidates the similarity in the community structure at depths >20 meters (the marked cluster).

Beta diversity analyses carried out for determining variations in bacterial community structure at various depths indicated that bacterial richness is more in higher depths i.e. >25 meters. Principal coordinate analysis (PCoA) carried out using Bray-Curtis metrics, showed that sediment samples from depths greater than 20 meters grouped together on PCoA plot (Fig 4). This indicates low intragroup variation in samples from 25–40 meters as compared to that in samples collected from depths 1–20 meters. Based on the PCoA analysis on bacterial diversity, we could group the samples as: upper continental shelf samples (UCS) with lesser depths i.e. 1–20 meters and lower continental shelf samples (LCS) with greater depths i.e. 25–40 meters. To confirm the inter group variation between UCS and LCS, additional statistical test like ANOVA (Tukey’s HSD) and Adonis were performed. Statistical analysis revealed that bacterial community structure between these groups is significantly different (p = 0.005, Adonis test). Additionally, comparison of phylum level diversity between UCS and LCS samples revealed significant differences in some of the phyla like *Acidobacteria* (p = 0.000) and *Chloroflexi* (p = 0.034) as mentioned in Table 2. Furthermore, significant differences were also observed in bacterial diversity belonging to families like *Flavobacteriaceae* (p = 1.716E-06), *Rhodobacteriaceae* (p = 4.92E-06) and *Vibrionaceae* (p = 0.005) between these two groups (Table 2).

### Imputed metagenomic functions of bacterial community

The marine environment is a diverse habitat harboring organisms from all hierarchies, right from prokaryotes to higher eukaryotes. Bacterial community analysis carried out in this study provided strong evidences that the continental shelf shelters diverse bacterial population. These findings created an urge to understand the functional diversity of the habitat. We used an approach of imputed metagenome using the tool PICRUSt [22] to determine bacterial gene pool across various depths of the continental shelf. The results obtained from this effort exhibited wide range of genetic diversity involved in various essential processes like environmental information signaling, signal transduction and metabolism of carbohydrates, proteins and other biomolecules including synthesis of some secondary metabolites.

### Mechanisms for nutrient acquisition

**Fig 5:**
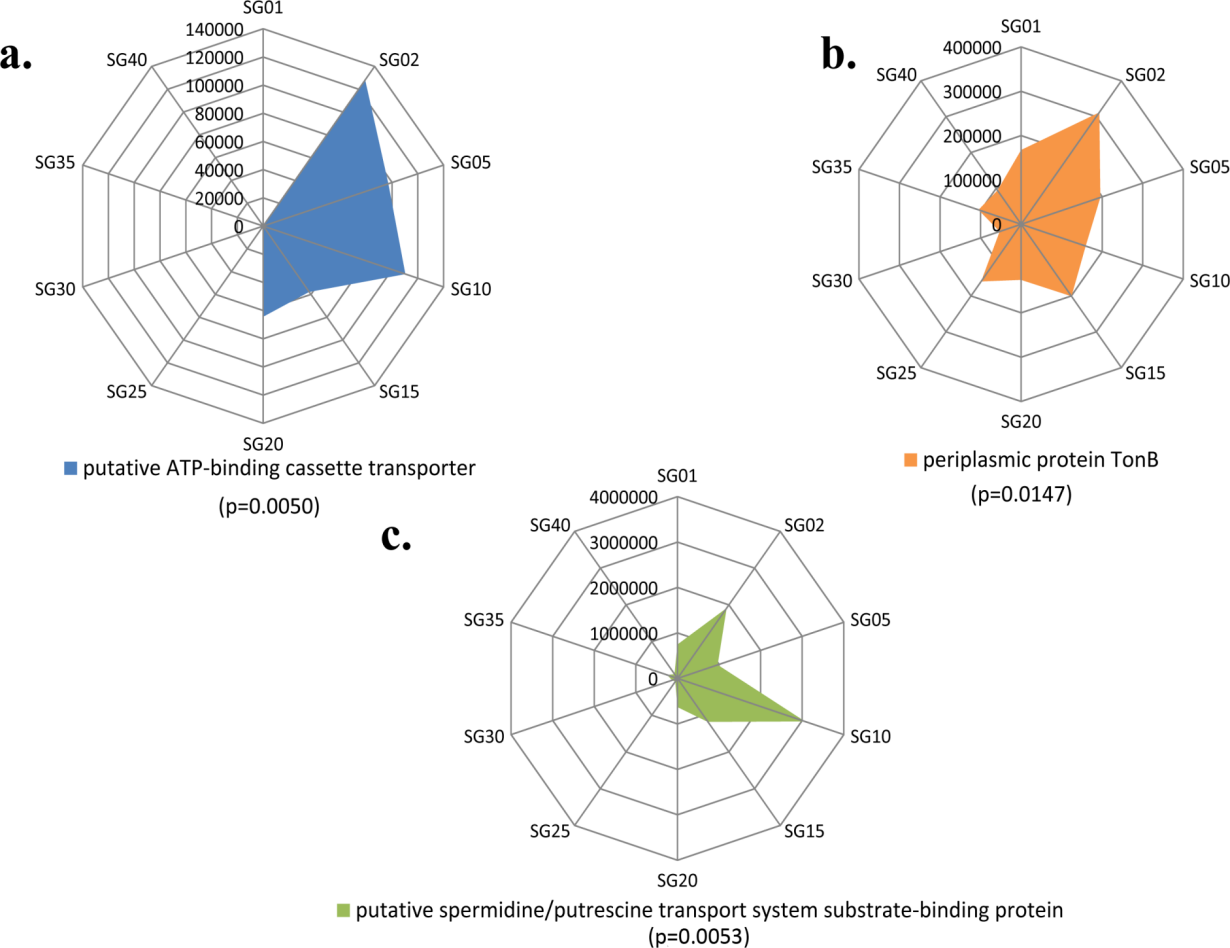
Distribution of functional genes across the samples. (a) Distribution of ABC transporter genes across the samples (p = 0.0050), showing a high abundance in the depths <20 meters. (b) Distribution of TonB protein coding genes, seen more abundant in the lower depths (p = 0.0147). (c) Distribution of polyamines coding genes across the samples, observed to be abundant in the depths < 20 meters (p = 0.0053).

Adaptation to acquire nutrients from surrounding environment is of prime importance to bacteria for their survival in the respective ecological niche [3,4]. Imputed metagenomic analysis carried out in this study provided substantial insights into such mechanisms in the continental shelf. One of the interesting observations was that, the bacterial communities from UCS harbored more genes involved in mechanisms of nutrient acquisition, as compared to LCS. These genes are involved in production of ATP binding cassette (ABC) transporters and periplasmic proteins like TonB (Fig 5). Previous reports have shown that production of these proteins is bio-energetically costly and are manufactured by bacteria in sufficient amounts, only during nutrient limiting conditions [3,4,37]. Additionally, it was also observed that genes encoding proteins for polyamine transport system were relatively abundant in UCS (Fig 5). Polyamine transport systems are basically involved in acquisition of polyamines like spermidine and putrescine, which are essential for cell survival [40,41]. They also have an additional role in the biofilm formation in some of the bacterial groups [41]. Biofilms are known to have viscoelastic property, which helps them to adhere and also to withstand the shear pressures in the environment [42]. So, this mechanism of acquisition of polyamines may play a key role in the biofilm formation, and thus provide stability to the bacterial community against the shear forces created due to the turbulence in the continental shelf. Also, previous metaproteomic studies on Sargasso Sea suggest that the transport (importers) systems like those involved in acquisition of polyamines are expressed in very high amounts in nutrient limiting conditions [3,4]. Apart from the mentioned genes, various other genes involved in transport systems of amino acid, taurine, simple sugars along with glutamine transport systems were found to be higher in the UCS (S3 Table). Higher abundance of these genes in the UCS indicates that, the upper continental shelf is a nutritionally deprived region (especially carbon depleted), thus harboring lower bacterial diversity with potential but bio-energetically costly adaptive mechanisms for nutrient uptake (S3 Table). Furthermore, our observations of distribution and diversity of functional genes across depths of the continental shelf, demonstrate the extent of bacterial adaptations to survive in the respective ecological niche.

### Biogeochemical cycles

Microbial communities are well known key players of biogeochemical cycles in the marine environment and majorly contribute to the global biogeochemical cycling of carbon and nitrogen [1,2,40]. Our effort of further mining imputed metagenomic data helped us in exploring microbial potential contributing to these vital processes in the continental shelf. Previous reports based on simulation models and some other basic observations suggest that nitrogen content of the shelf is much higher than total oceanic nitrogen content [1]. The genes involved in denitrification processes were found in almost all depths, being more abundant in LCS (*nitrite reductase*, p = 0.03). Genes involved in denitrification process help microbes to contribute remarkably in ceasing nitrogen flux from land to ocean and thus acting as a biological barrier [1]. Similarly, organic matter utilizing genes instrumental in carbon cycle were also observed. Thus, these genes involved in nitrogen and carbon cycling contribute in driving the biological pump [2,40]. Furthermore, interesting observations were made regarding the systems involved in assimilation of methanol, which is known to be one of the important substrates for bacterioplanktons [3,43]. The presence of genes encoding methanol dehydrogenase in bacterial communities of the continental shelf explains the wide potential of these microbes to utilize single carbon compounds. Additionally, various genes involved in reverse methanogenesis were also detected throughout the continental shelf. These included genes required in the production of a set of enzymes like *methenyltetrahydrofolate cyclohydrolase* [EC: 3.5.4.9] and *methylene-tetrahydromethanopterin dehydrogenase* [EC: 1.5.98.1], which are part of a pathway for anaerobic oxidation of methane [44]. This pathway is prevalent in anaerobic methanotrophic archaea, as reported in previous studies [3,4]. It was noticed that bacteria in the continental shelf also harbor gene pool of ‘Phn’ proteins (*phnB*, *phnG*, *phnH*, *phnI*, *phnM*, *phnO*, *phnP*), which are part of a C-P (Carbon-Phosphorus) lyase pathway [40,42]. The ‘Phn’ proteins that form carbon-phosphorus lyase complex are involved in production of methane from phosphorus containing organic substrate called methylphosphonate [45]. This observation indicates that bacteria present in the continental shelf contribute largely to the aerobic production of the green-house gas. Apart from these genes, as mentioned previously the ABC transporters contribute largely to the cycling of DOC (Dissolved Organic Carbon) in the ocean which involves both importers and exporter proteins. List and description of other genes involved in biogeochemical cycling and those important in nutrient acquisition is provided as supplementary information (S3 Table).

In summary, remarkable differences were observed in bacterial diversity within a short region of the continental shelf (1–40 meters) along with diverse metabolic potential. To the best of our knowledge, this is the first report of determining bacterial diversity and composition from Agatti Island (Lakshadweep Archipelago). Our findings exhibit the importance of several adaptive mechanisms developed by the bacterial community to survive in nutritionally deprived conditions, and also help us to understand the influence of nutrition availability on bacterial diversity. Overall, our effort of carrying out the imputed metagenome analysis, gave deeper insights into the functional aspects of bacterial community, which is imperative in any ecological study. From the observations of extensive adaptive mechanisms and vast array of biological pathways involved, it is clear that, much has to be explored from such an important biological hotspot. Our study is a step forward in understanding the widespread bacterial diversity of the marine environment, which can serve as an elementary data to several future multi-omics studies aiming to understand the ecology of marine habitats.

## Supporting Information

S1 Fig Phylogenetic tree analysis. The tree was constructed using Neighbor-Joining (NJ) method. Bootstrap values only greater than 80 are displayed in the tree.
(PDF)

S1 Table Cultured diversity. Taxonomic classification of the bacterial isolates, along with the sequence similarity (%) with their respective closest hit from EzTaxon database.
(XLSX)

S2 Table Relative abundance. The table depicts the relative abundance (in %) of total bacterial community assigned to different phyla.
(XLSX)

S3 Table Metagenomic predictions. The table consists of results of the imputed metagenomic predictions along with the OTU ID’s and KEGG annotations.
(XLSX)
