# Supplementary figures and images for: Insights into Diversity and Imputed Metabolic Potential of Bacterial Communities in the Continental Shelf of Agatti Island
Source: PLoS One. 2015 Jun 11;10(6):e0129864. doi: 10.1371/journal.pone.0129864 (PMC4465901; doi:10.1371/journal.pone.0129864)

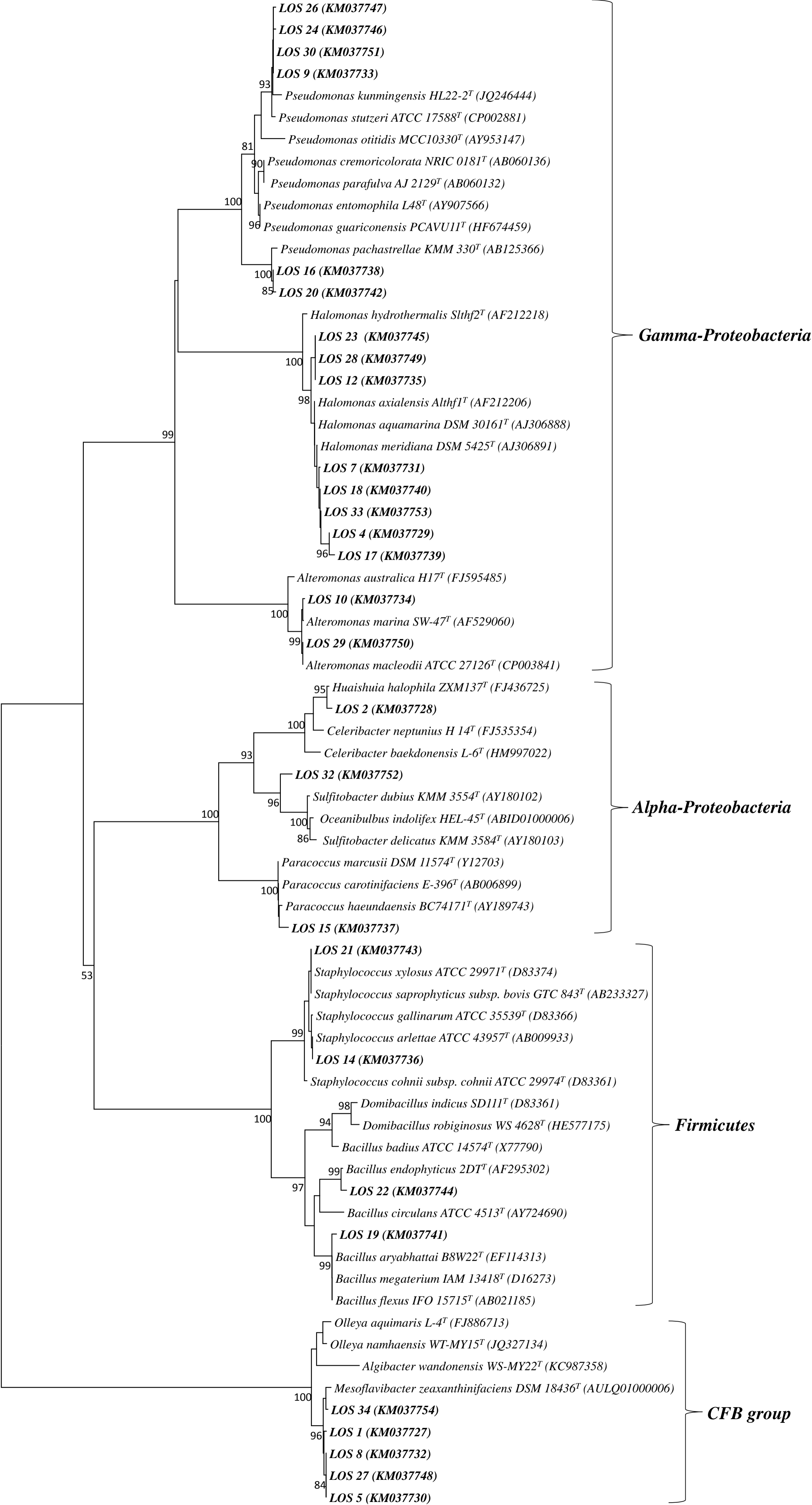

0.05

Supplement: S1 Fig — The tree was constructed using Neighbor-Joining (NJ) method. Bootstrap values only greater than 80 are displayed in the tree. (PDF) [file pone.0129864.s001.pdf]
